# Assessment of anticancer, antimicrobial, antidiabetic, anti-obesity and antioxidant activity of *Ocimum Basilicum* seeds essential oil from Palestine

**DOI:** 10.1186/s12906-023-04058-w

**Published:** 2023-07-04

**Authors:** Ahmad M Eid, Nidal Jaradat, Naser Shraim, Mohammed Hawash, Linda Issa, Mohammad Shakhsher, Nour Nawahda, Ali Hanbali, Noor Barahmeh, Basil Taha, Ahmed Mousa

**Affiliations:** 1 Department of Pharmacy, Faculty of Medicine and Health Sciences, An-Najah National University, P.O. Box 7, Nablus, Palestine; 2 Department of Biomedical Sciences, Faculty of Medicine and Health Sciences, An-Najah National University, Nablus, Palestine

**Keywords:** *Ocimum basilicum*, Anticancer, Antimicrobial, Antioxidant, Anti-diabetic, Anti-obesity

## Abstract

**Background:**

Many modern pharmaceutical researchers continue to focus on the discovery and evaluation of natural compounds for possible therapies for obesity, diabetes, infections, cancer, and oxidative stress. Extraction of *Ocimum basilicum* seed essential oil and evaluation of its antioxidant, anti-obesity, antidiabetic, antibacterial, and cytotoxic activities were the goals of the current study.

**Method:**

*O*. *basilicum* seed essential oil was extracted and evaluated for its anticancer, antimicrobial, antioxidant, anti-obesity, and anti-diabetic properties utilizing standard biomedical assays.

**Results:**

*O*. *basilicum* seed essential oil showed good anticancer activity against Hep3B (IC_50_ 56.23 ± 1.32 µg/ml) and MCF-7 (80.35 ± 1.17 µg/ml) when compared with the positive control, Doxorubicin. In addition, the essential oil showed potent antibacterial (against *Klebsiella pneumoniae, Escherichia coli, Staphylococcus aureus, Proteus mirabilis*, and *Pseudomonas aeruginosa*) and antifungal (against *Candida albicans*) activities. Moreover, as for the anti-amylase test, IC_50_ was 74.13 ± 1.1 µg/ml, a potent effect compared with the IC_50_ of acarbose, which was 28.10 ± 0.7 µg/ml. On the other hand, for the anti-lipase test, the IC_50_ was 112.20 ± 0.7 µg/ml a moderate effect compared with the IC_50_ of orlistat, which was 12.30 ± 0.8 µg/ml. Finally, the oil had a potent antioxidant effect with an IC_50_ of 23.44 ± 0.9 µg/ml compared with trolox (IC_50_ was 2.7 ± 0.5 µg/ml).

**Conclusion:**

This study has provided initial data that supports the importance of *O. basilcum* essential oil in traditional medicine. The extracted oil not only exhibited significant anticancer, antimicrobial, and antioxidant properties but also antidiabetic and anti-obesity effects, which provided a foundation for future research.

## Introduction

Herbal medicine is still practiced today due to its biomedical benefits as well as cultural beliefs in many parts of the world [1]. In Palestine, traditional herbal therapy has a long history of usage and continues to provide significant benefits in the treatment of many disorders [2]. However, scientific explorations of the therapeutic and chemical qualities of plants utilized in traditional Palestinian medicine are trivial [3]. According to reports, a number of traditional Palestinian medicinal herbs possess remarkable bioactivities that may contribute to the enhancement of community health and lifestyle. Since ancient times, people have relied on the healing and preservation powers of medicinal aromatic herbs [4, 5]. Essential oils, which are secondary metabolites of plants, are responsible for some of these activities [6]. These substances have the physical aim of protecting plants against bacteria, fungi, viruses, insects, and even herbivores by lowering their desire for these plants [7]. Antimicrobial, sedative, food additive, and cosmetic uses for essential oils are just a few of the many essential oil based products on the market [7–9]. The result is that a number of research teams are investigating the fundamental components of essential oils and their antioxidant, anthelmintic, and antibacterial properties, as well as the primary components of essential oils and their antifungal properties [10–12].

Oxidative stress causes reactive oxygen species (ROS), which are known to contribute to a number of cardiovascular disorders, including heart failure, atherosclerosis, and ventricular remodeling. Antioxidants and pro-oxidants may have contradictory effects on the development of cancer; they can prevent oxidative stress to DNA and inhibit tumorigenesis [13]. It is the usual cellular signaling pathways that are interrupted when this delicate equilibrium is upset, resulting in unchecked cell multiplication. Due to the overproduction of reactive oxygen, oxidative stress plays a vital role in atherosclerotic lesion formation. Through the activity of several enzymes, endothelial cells and smooth muscle cells are capable of producing oxidants. The majority of reactive oxygen in the vascular wall is generated by NADPH oxidases, a group of membrane-associated enzymes typical of cells of mesodermal origin [13, 14]. A greater level of oxidative stress is seen in these situations and in the tumor environment, so the increased antioxidant system can take advantage of the upregulated antioxidant system [13, 15]. A number of antioxidants, including coenzyme Q10, beta-carotene, lycopene, quercetin, and vitamins C and E, have been found to be effective in the prevention and treatment of various forms of cardiovascular disease (CVD) [13].

Worldwide, the prevalence of obesity has roughly tripled since 1975, making it a growing health concern. There has been an increase in obesity in low- and middle-income nations, particularly in metropolitan areas, since the 1970s [16]. Overweight and obese children in Palestine accounted for 6% of the population in children and 18% of the population in adults, respectively [17]. Increased BMI is connected with non-communicable diseases such as cardiovascular disease, diabetes, and numerous types of cancer [18]. Along with obesity, diabetes mellitus and its consequences are serious health issues that pressure the healthcare system and people. The frequency is rising worldwide, especially in low- and middle-income nations. Approximately 422 million people globally have diabetes, according to a recent estimate [19]. In Palestine, diabetes prevalence was estimated in 2000 at 9.7%, rising to 15.3% by 2010, and it is anticipated to reach 20.8% by 2020 and 23.4% by 2030 for persons aged 25 and over in the country [20].

The biggest global risk to effectively treating infections caused by harmful pathogens right now is antimicrobial resistance. Antimicrobial agent resistance has been shown to have negative effects on both clinical and therapeutic outcomes, with costs ranging from longer hospital stays, higher rates of morbidity and mortality, and more expensive healthcare to treatment failures and the need for more expensive and safe alternative medications. In the ongoing fight against microbial infections, there is an urgent need for new antimicrobial agents. Antimicrobial resistance is unavoidable, and pharmaceutical firms consistently show little interest in funding novel antibiotic research [21–24]. The abuse and misuse of antimicrobials has led to an increase in the number of “antibiotic-resistant bacteria,“ which can no longer be treated with current antimicrobials because of the rise in antimicrobial resistance. To combat the rise of antibiotic-resistant bacteria and the negative effects of synthetic antimicrobials, researchers have turned to natural remedies [25].

**Fig. 1:**
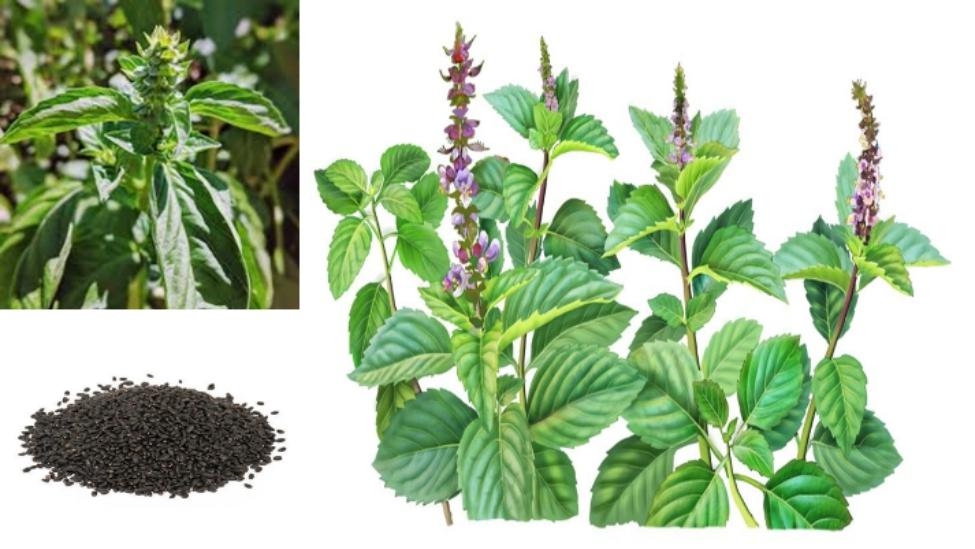
Basil (*Ocimum basilicum*) plant

Great basil, also known as Saint-Joseph’s wort or *Ocimum basilicum* (*O. basilicum*), is a major member of the *Lamiaceae* family, which is often known as the mint family [26, 27]. Previous scientific studies revealed many pharmacological effects in curing several health problems, this plant showed potent antioxidant, anticancer, antiviral, anti-aging, and antimicrobial properties [28–30]. *O. basilicum* seeds can be described as tiny black, ellipsoid seeds, as shown in Fig. 1 [31]. These seeds are widely used in traditional medicine to treat colic ulcers, dyspepsia, and diarrhea [1, 32]. *O. basilicum* seeds have a remarkable capacity for hydration because of their adherent seed mucilage, which is known to be produced in testa cells during seed development [33, 34]. Research has shown that rosmarinic acid is the main biologically active component in *O. basilicum* that is related to these activities [35].

As previously noted, basil has been shown to help digestion, and when combined with camphor, it can also be used to treat epistaxis. An internal infusion of *O. basilicum* is used to treat a variety of ailments, including cephalalgia, high fever, cough, gouty joint pain, otitis media, snake bites, gastroenteritis, and piles. gonorrhea, chronic dysentery, diarrhea, and diabetes mellitus can be treated with an infusion of basil seed [36, 37]. This research focused on the study of the anticancer, antimicrobial, antioxidant, anti-obesity, and antidiabetic effects of the *O. basilicum* essential oil since no previous studies have investigated these healthcare problems with the *O. basilicum* seeds essential oil from Palestine.

## Materials and methods

### Chemical reagents and instruments

2,2-Diphenyl-1-picrylhydrazyl (DPPH) (Sigma Aldrich, Germany) and ((s)-(-)-6 hydroxy-2,5,7,8-tetramethychroman-2-carboxylic acid) (Sigma Aldrich, Denmark). n-hexane (Frutarom Ltd, Israel). Methanol (Lobachemie, India), The solvents used for the extraction of plant material were of HPLC grade. Sigma-Aldrich (USA) provided porcine pancreatic lipase, starch, acarbose, DPPH (2,2-diphenyl-1-picrylhydrazyl), PNPP (p-nitrophenyl palmitate), and orlistat, while MP Biochemicals (Illkirch, France) provided porcine pancreatic α-amylase. All the chemicals used in the experiments were of analytical grade. *O. basilicum* seeds were obtained from different regions in Palestine. The plant seeds were characterized in the Pharmacy Department at An-Najah National University. The plant material was collected in accordance with the WHO Guidelines for the Assessment of Herbal Medicines and Legislation.

The rotary evaporator (Heidolph OB2000, VV2000, Germany), the grinder (Moulinex model, Uno, China), the balance (Radwag, AS 220/c/2, Poland), and the filter papers (Machrery-Nagel, MN 617, Whatman no. 1, USA) were all used.

### Collection and extraction of ***O***. ***basilicum*** essential oil

The seeds of the plant were gathered from various areas in Palestine and then processed into a fine powder using a mechanical blender. This yielded 25 g of powdered plant material, which was then extracted utilizing the hydrodistillation Clevenger technique for 4 h based on the method described in the British Pharmacopoeia. The resulting oil was chemically dried by utilizing calcium carbonate and stored at 4–8 °C in a dark bottle until further use [38].

### Antioxidant activity of ***O. basilicum*** essential oil in terms of free radical scavenging

At first, the *O. basilicum* essential oil and the Trolox standard were mixed together to make a stock solution with a concentration of 1 mg/ml that was dissolved in methanol. Stock solutions were used to prepare working solutions with the following concentrations (2, 3, 5, 10, 20, 30, 50, 80, and 100 µg/ml) by serial dilution in methanol. The following concentrations were achieved by diluting stock solutions sequentially in methanol. A newly made solution of DPPH with a concentration of 0.002% weight-per-volume was created. After that, it was combined with methanol and each of the working concentrations in a proportion of 1:1:1, starting with the lowest concentration. Methanol was used as a blank solution for the spectrometer assay for the DPPH assay. The DPPH solution mixed with just methanol was the starting point for the series of concentrations. Around thirty minutes were spent incubating the solutions at room temperature in a completely dark cabinet. The absorbance of the samples was then measured with the spectrophotometer set to a wave length of 517 nm [39].

Using the following formula, we were able to determine the proportion of antioxidant activity contained within the extracted oil in comparison to the Trolox standard:

DPPH inhibition activity (%) = (A-B)/A ×100%.

Where A is the absorbance of the blank and B is the absorbance of the sample. The data was analyzed and reported as the percentage of inhibition. The inhibition of the extracted oil and the Trolox standard at different concentrations, were plotted and tabulated.

### Antidiabetic evaluation of ***O. basilicum*** essential oil using the α-amylase inhibitory method

Following a scientifically adopted protocol in the enzymatic assay of amylase inhibitory action [40], the *O*. *basilicum* essential oil was dissolved in a few milliliters of 10% DMSO and mixed with buffer (0.02 M Na_2_HPO4/NaH_2_PO_4_, 0.006 M NaCl, pH 6.9). Different dilutions were prepared (50, 100, 200, 400, and 1000 µg/ml) using 10% DMSO as the solvent for the dilution. Then, 0.2 ml of porcine pancreatic-amylase enzyme solution (2 units/ml) was prepared and mixed with 0.2 ml of *O. basilicum* essential oil before incubating this sample for 10 min at 30 •C. Following the previous step, 0.2 ml of the freshly prepared 1% starch aqueous solution was incubated for 3 min and then added to each tube. To stop the reaction after diluting each sample by 5 ml of distilled water, 0.2 ml of DNSA solution was then added. Finally, each tube was warmed at 90 •C in a water bath for 10 min and then allowed to cool down, with the spectrophotometer adjusted after that at 540 nm to take absorbance for each tube. The blank was made using the same previous steps without adding the essential oil. As a standard reference, acarbose was used with the same previous steps for comparison as a standard.

We used the formula: amylase inhibition% = (Ab - As)/Ab * 100%.

Ab: blank absorbance, As: sample absorbance.

### Anti-obesity evaluation ***O. basilicum*** essential oil using the lipase inhibitory method

The porcine pancreatic lipase inhibitory experiment was performed on *O. basilicum* essential oil using modified methods from Zheng et al. (2010) and Bustanji et al. (2010) [16, 17]. At the beginning, a stock solution of *O. basilicum* essential oil in 10% DMSO at a concentration of 1 mg/ml (1000 µg/mL) was employed. From this solution, five different solutions with the following concentrations were prepared: 50, 100, 200, 300, and 400 µg/ml respectively. Just before its application, a stock solution of pancreatic lipase enzyme at a concentration of 1 mg/ml was made. To make a stock solution of PNPB (p-nitrophenyl butyrate), 20.9 mg of PNPB were dissolved in 2 mL of acetonitrile. This produced the stock solution. In test tubes that already contained 0.2 mL of *O. basilicum* at one of five different concentrations (50, 100, 200, 300, or 400 µg/ml), 0.1 ml of porcine pancreatic lipase at a concentration of 1 mg/mL was added. After that, the resultant mixtures were diluted to a volume of 1 ml with a Tri-HCl solution (pH 7.4) and left to incubate at a temperature of 25 •C for 15 min. When the period of incubation had concluded, 0.1 milliliter of PNPB solution was poured into each test tube. The combination went back into the incubator for another half an hour at 37 •C. An UV-visible spectrophotometer was used to evaluate the level of pancreatic lipase activity by monitoring the rate at which p-nitrophenyl butyrate was converted into p-nitrophenol at a wavelength of 405 nm. The exact same process was carried out using orlistat, which served as a positive control, utilizing the identical concentrations that were described before.

We used the formula: lipase inhibition% = (Ab - As)/Ab * 100%.

Ab: blank absorbance, As: sample absorbance.

### Antimicrobial evaluation of ***O. basilicum*** essential oil

The antibacterial activity of *O. basilicum* essential oil was tested using five strains obtained from the American Type Culture Collection (ATCC), including *Klebsiella pneumoniae*, *Escherichia coli*, *Staphylococcus aureus*, *Proteus mirabilis*, and *Pseudomonas aeruginosa*, in addition to the clinical isolate Methicillin-Resistant *Staphylococcus aureus* (MRSA). For the antifungal test, *Candida albicans* was employed.

The essential oil of *O. basilicum* has been tested for antimicrobial activity using the broth microdilution technique. *O. basilicum* essential oil stock solution was prepared with a concentration of 200 µg/mL 20%, dissolved in 20% DMSO and 60% Muller-Hinton broth. The prepared *O. basilicum* essential oil solution was serially diluted (2-folds) with sterile Muller-Hinton broth to achieve serial dilutions of 50, 25, 12.5, 6.25, 3.125, etc. µg/mL (RPMI medium was used for the *C. albicans* strain); DMSO concentration was 5% at the first well and was then serially diluted two-fold to exclude its antimicrobial effect. In 96-well plates, the dilution procedure was conducted aseptically. Micro-well 11 contained *O. basilicum* essential oil-free media (inoculated with the microbe), which served as a positive control for microbial growth in the micro-wells used to evaluate the antibacterial activity of the essential oil. On the other hand, the Mueller-Hinton broth in microwell 12 was oil- and microbial-free, which serves as a negative control for the proliferation of microorganisms. There were no microbes found in this well when it was utilized as a negative control. The test microorganisms were injected into micro-wells 1–11. The antibacterial activity of *O. basilicum* essential oil was evaluated three times. All inoculation plates were kept at 35 •C. Plates containing test bacterial strains were incubated for approximately 18–24 h, whereas plates containing *C. albicans* were incubated for approximately 48 h. When no microbial growth could be seen in the micro-wells after adding a modest dose of *O. basilicum* essential oil, the MIC was determined to be that concentration. It was decided to use antimicrobial drugs with established antibacterial activity to test the approach with the microorganisms, such as ampicillin and ciprofloxacin for bacteria and fluconazole for molds [40].

### Cytotoxicity assay of ***O. basilicum*** essential oil

A cytotoxicity assay was performed for *O. basilicum* essential oil and doxorubicin (Positive control). HeLa, MCF-7, and Hep3B cells were grown in RPMI-1640 medium (Sigma-Norwich, United Kingdom) that was supplemented with 1% L-glutamine (Sigma, United Kingdom), 1% penicillin/streptomycin antibiotics (BI, India), and 10% fetal bovine serum. It was kept at 37 •C in a humidified environment with 5% CO_2_ in order to develop cancer cells. In a 96-well plate, cells were planted at a density of 2.6104 cells per well. *O. basilicum* essential oil doses of 300, 120, 60, 30, and 10 µg/ml were incubated for 24 h with cancer cells after 48 h of incubation with the tested *O. basilicum* essential oil. The CellTilter 96® Aqueous One Solution Cell Proliferation (MTS) Assay was used to determine the viability of the cells in accordance with the manufacturer’s instructions (Promega Corporation, Madison, USA). MTS solution was added to each well after the treatment, and the well plates were incubated at 37 •C for two hours. Four hundred and ninety-nine-nanometer absorbance. The same procedure was carried out for doxorubicin (positive control) [39].

### Data analysis

The cytotoxic, antimicrobial, antioxidant, anti-obesity, and antidiabetic activities were tested for *O. basilicum* essential oil and were performed in triplicate. The data were presented as the mean and standard deviation (± SD). The results were considered significant only when the p values were less than 0.005.

## Results

### Antioxidant activity of ***O. basilicum*** essential oil

**Fig. 2:**
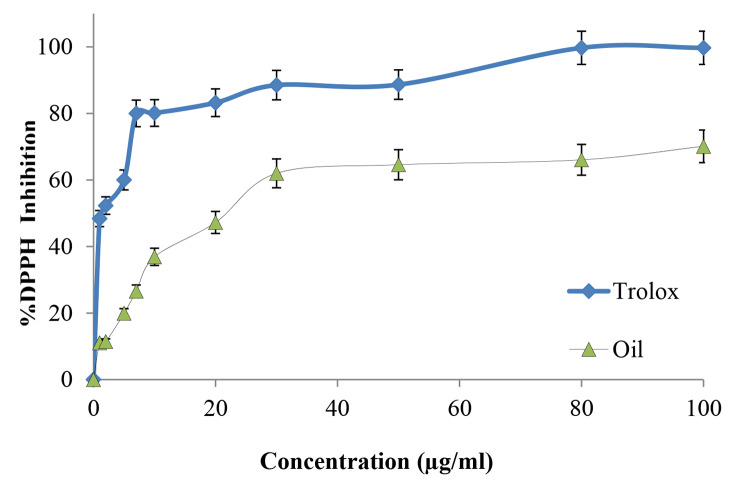
%DPPH inhibition profile by trolox and *O. basilicum* essential oil

The free radical scavenging activity of the extracted *O. basilicum* essential oil was tested by the DPPH radical method using trolox as a reference standard and shown in Fig. 2. In this study, *O. basilicum* essential oil showed good antioxidant activity (IC_50_ = 23.44 ± 0.9 µg/ml) when compared to the standard antioxidant compound trolox (IC_50_ = 2.7 ± 0.5 µg/ml).

### Antidiabetic activity of ***O. basilicum*** essential oil

**Fig. 3:**
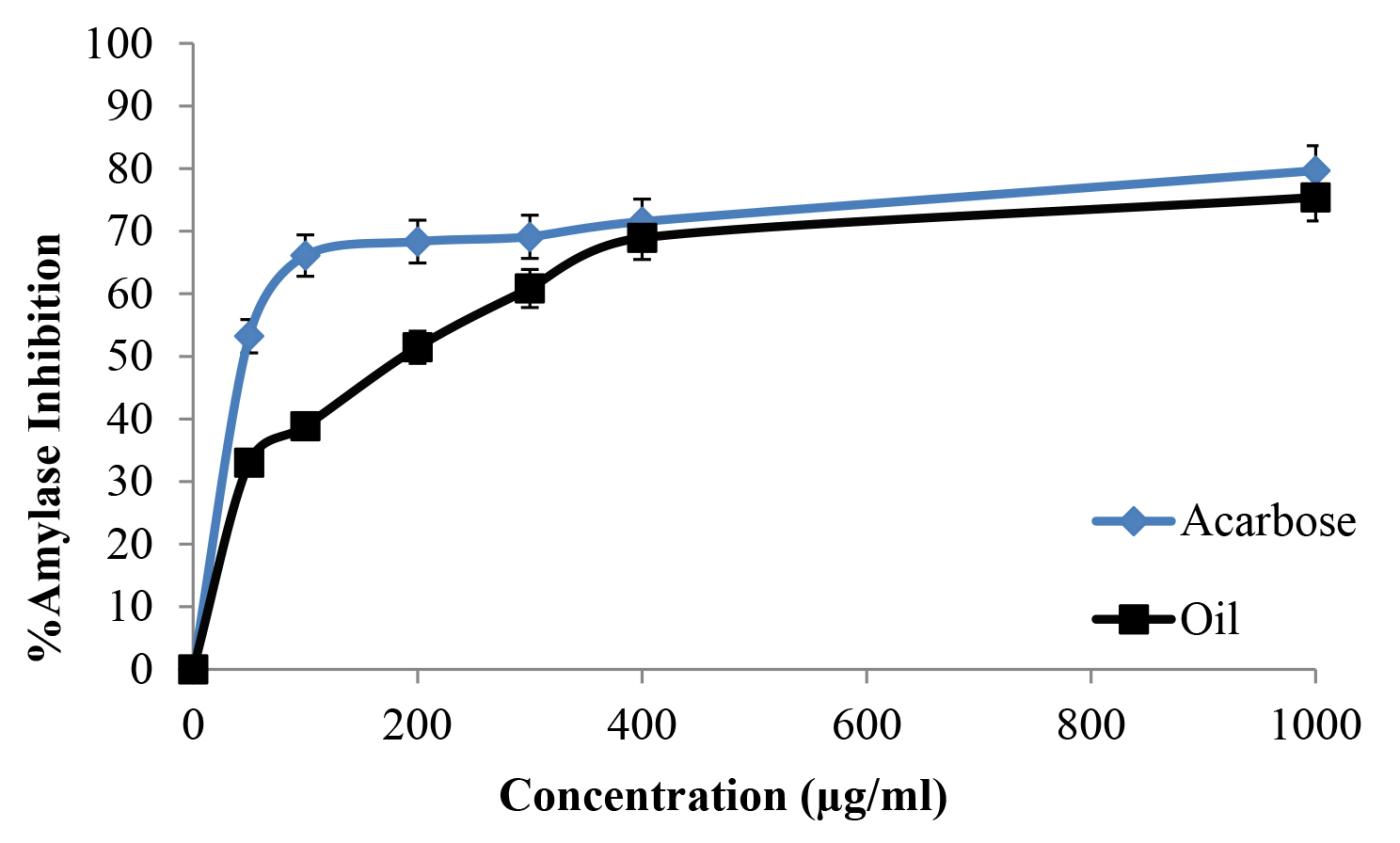
α-Amylase inhibitory activity profile by acarbose standard and *O. basilicum* essential oil

The α-amylase inhibitory activity of the extracted *O. basilicum* essential oil was evaluated against porcine pancreatic α-amylase. The result is shown in Fig. 3. When compared to the standard acarbose (IC_50_ = 28.10 ± 0.7 µg/ml), *O. basilicum* essential oil demonstrated strong α -amylase inhibitory activity with an IC_50_ = 74.13 ± 1.1 µg/ml.

### Anti-obesity activity of ***O. basilicum*** essential oil

**Fig. 4:**
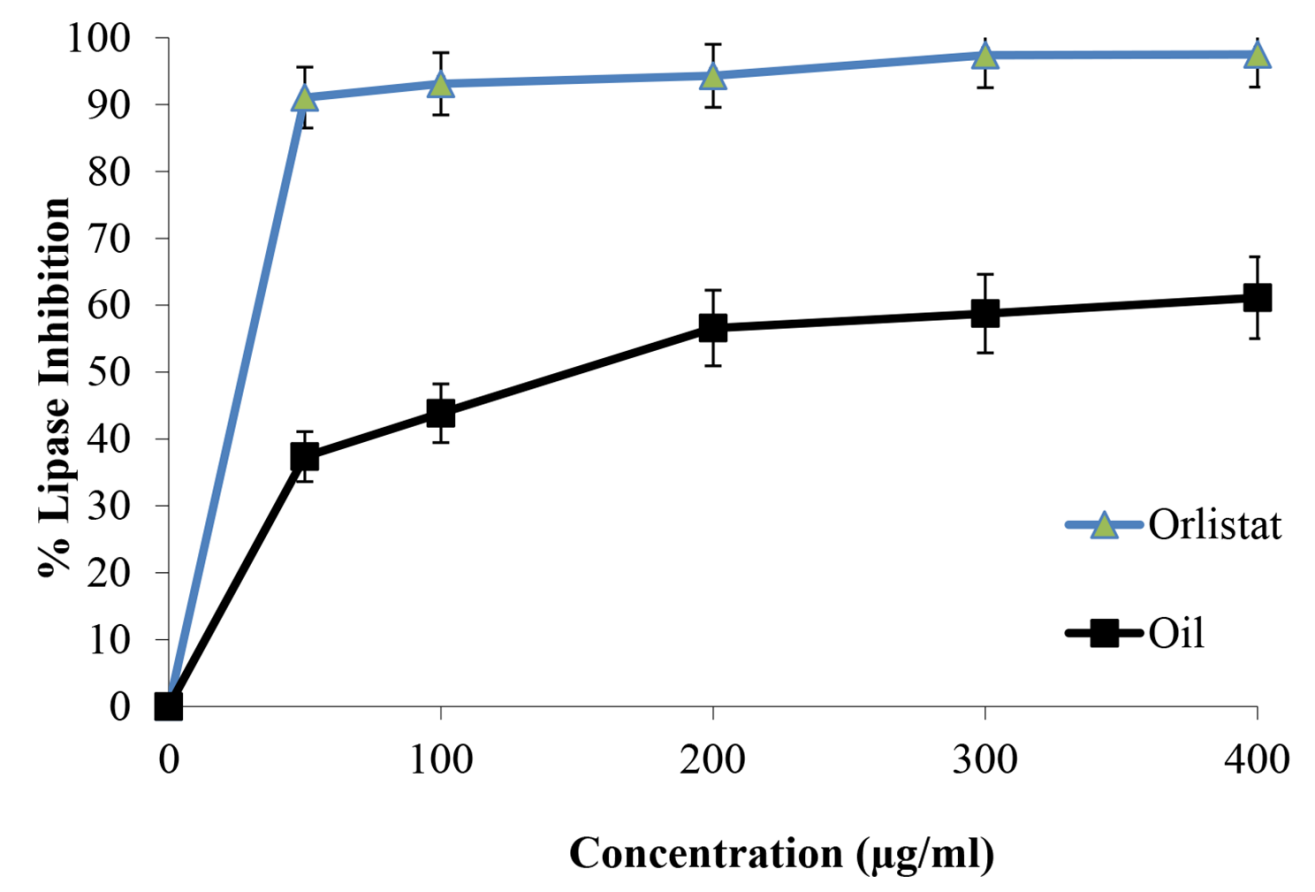
Porcine pancreatic lipase inhibitory activity profile by orlistat standard and *O. basilicum* essential oil

The porcine pancreatic lipase inhibitory activity of the extracted *O. basilicum* essential oil was evaluated and shown in Fig. 4. The results revealed that *O. basilicum* essential oil has moderate to good activity against porcine pancreatic lipase enzyme (IC_50_ = 112.20 ± 0.7 µg/ml) when compared with the orlistat standard (IC_50_ = 12.30 ± 0.8 µg/ml).

### Antimicrobial activity of ***O. basilicum*** essential oil

The antibacterial test of *O. basilicum* essential oil was performed on different strains of gram-positive and gram-negative bacteria using broth microdilution and showed broad-spectrum antibacterial and antifungal effects against all the screened bacterial and fungal strains in comparison with control positive antibiotics and antifungals, such as ampicillin, ciprofloxacin, and fluconazole, respectively. The *O. basilicum* essential oil MIC values are demonstrated in Table 1.

**Table 1:** MIC values (µg/mL ± 0.05) of *O. basilicum* essential oil compared with Ampicillin, Ciprofloxacin, and Fluconazole antibiotics,

Microorganisms	*O. basilicum* oil	Ampicillin	Ciprofloxacin	Fluconazole
***S. aureus*(**ATCC 25,923)	2.3	6.25	0.78	-
**MRSA**	2.3	32	12.5	-
***E. coli*** (ATCC 25,922)	1.0	3.12	0.78	-
***P. vulgaris*(**ATCC 8427)	1.5	3.25	0.06	-
***K. pneumonia*** (ATCC 13,883)	1.0	12.5	0.06	-
***P. aeruginosa*** (ATCC 9027)	1.0	100	3.12	-
***C. albicans*** (ATCC 90,028)	1.3	-	-	3.12

### Cytotoxicity effect of ***O. basilicum*** essential oil

**Fig. 5:**
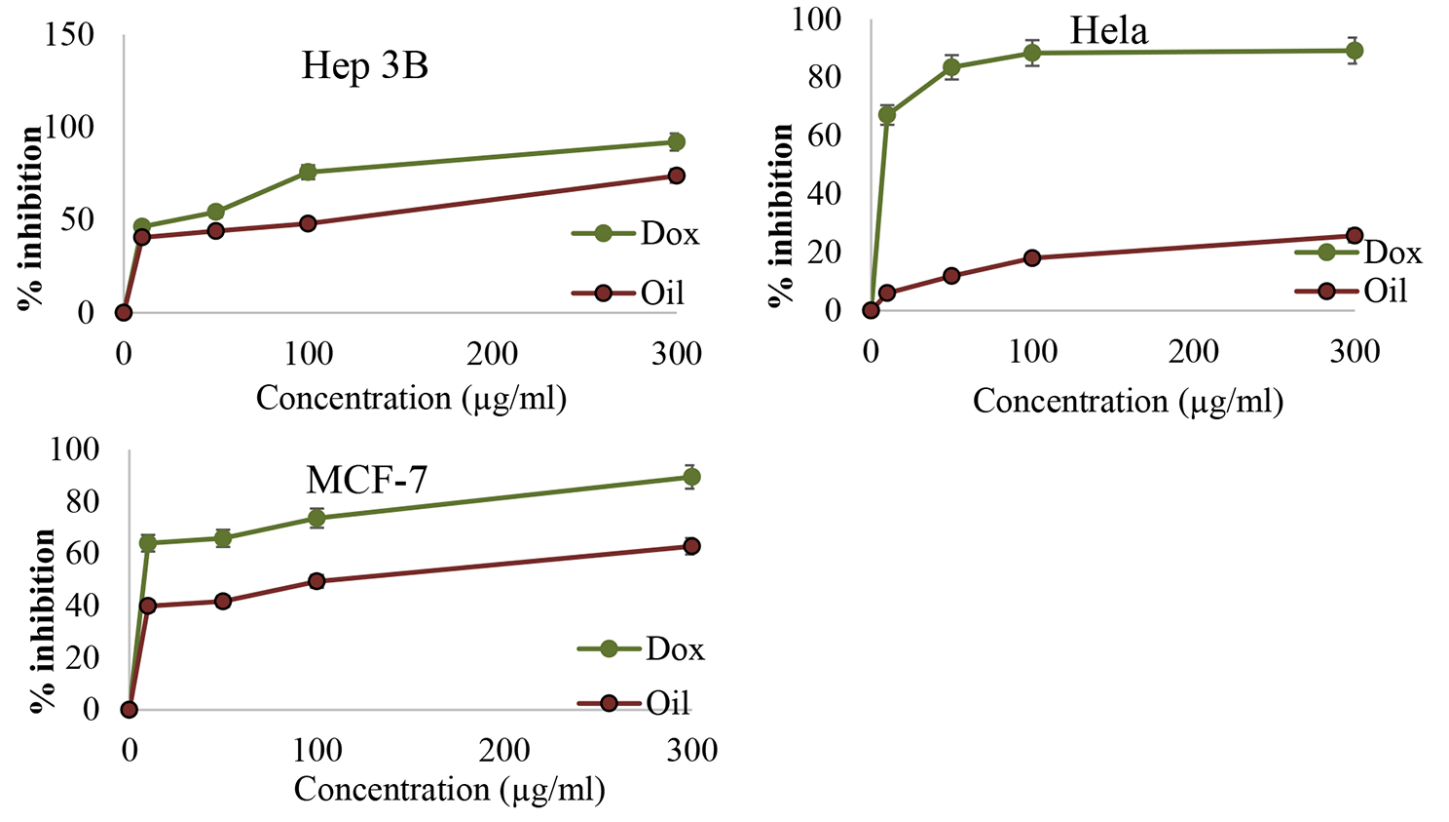
The IC_50_ values (µg/mL) of *O. basilicum* essential oil and Doxorubicin against different cancer cells line

After treatment of HeLa, MCF-7, and Hep3B tumor cells with *O. basilicum* essential oil, the MTS assay results showed an interesting result (Fig. 5), which explains the relationship between the concentration of *O. basilicum* essential oil and doxorubicin plotted against the inhibition percent of cancer cell growth. When the concentrations of oil and doxorubicin increased, the inhibition of the growth of cancer cells also increased, which means that there is an effect of oil against these cancer cells. *O. basilicum* essential oil had a good cytotoxic effect against MCF-7 and Hep3B cancer cell lines that showed an IC_50_ value of 80.35 ± 1.17 µg/ml and 56.23 ± 1.32 µg/ml respectively. However, its activity against Hela was inactive. These results were compared with the IC_50_ of doxorubicin, as shown in Table 2.

**Table 2:** The IC_50_ values (µg/mL) of *O. basilicum* essential oil and Doxorubicin against different cancer cells line

	Hep 3B	Hela	MCF-7
***O. basilicum*** **essential oil IC**_**50**_ **(µg/ml)**	56.23 ± 1.32	Inactive (53.70 mg/ml)	80.35 ± 1.17
**Doxorubicin IC**_**50**_ **(µg/ml)**	21.37 ± 0.62	10.11 ± 1.17	15.02 ± 0.72

## Discussion

The well-known *O. basilicum* (basil plant) includes a number of bioactive secondary metabolites, including polyphenols, flavonoids, and terpenes, which have the potential to have a wide range of biological effects. Its essential oil is mainly composed of linalool, methyl estragole, methyl cinnamate, and methyl chavicol [41, 42]. The fact that there are over 25 distinct varieties of *O. basilicum*, each with a unique constitution, has made it hard for previous researchers to create a uniform fundamental composition for basil extract or oil [43]. Since the DPPH radical is stable in its radical state, it is one of the most frequently utilized substrates for rapid evaluation of antioxidant activity. It was determined whether or not the extracts studied may function as hydrogen or electron donors in the transformation of DPPH into its reduced form DPPH-H by the use of the DPPH assay [44]. DPPH is a free radical that may be used to evaluate the effectiveness of pharmaceutical or herbal items to scavenge free radicals. Following both electron transfer and hydrogen atom transfer processes, it provides an evaluation of antioxidants that includes the ability to follow any or both of these processes [45]. The *O. basilicum* essential oil in the present work showed significant power to scavenge free radicals with a potent antioxidant effect, having an IC_50_ 23.44 ± 0.9 µg/ml compared with trolox (IC_50_ was 2.7 ± 0.5 µg/ml). *O. basilicum* essential oil demonstrated a higher content of phenols and maximal levels of DNA protection and free radical scavenging against toxicity induced by cadmium chloride [46]. As found in other research, the antioxidant property of this plant oil may be due to the polyphenoid rosmarinic acid, which is a derivative of cinnamic acid [47].

Health problems such as diabetes and obesity are becoming more prevalent throughout the globe [48]. They have been linked to an increase in the mortality rates of cancer, cardiovascular, respiratory, hepatic, and renal illnesses in several studies [49]. A high frequency of these metabolic illnesses has been evident and alarming for the past three decades in Palestine and many other nations worldwide [50]. To put it another way, any pharmacological drug that interferes with the activity of metabolic enzymes like lipase and amylase is guaranteed to be effective in curing these life-threatening conditions. Aromatic herbs have been utilized for a long time to cure obesity, overweightness, dyslipidemia, and diabetes in several global medical systems [51].

According to previous research findings, *O. basilicum* inhibits human pancreatic α-amylase in the small intestine, suppressing carbohydrate digestion and thus controlling the entry of glucose into the human body to maintain a reasonable postprandial glucose level [52–54]. Malapermal et al. examined the α-amylase inhibitory activity of aqueous, 60% ethanolic, and 70% ethanolic extracts of the leaves of *O. basilicum*, and the extracts exhibited nearly equivalent efficacy to acarbose [55]. Noor et al. evaluated the anti-diabetic potential of *O. basilicum* using porcine pancreatic α-amylase inhibitory activities. The results revealed that *O. basilicum* exhibited α-amylase inhibitory activity almost equivalent to the drug acarbose [54]. As we obtained in this study, the anti-amylase test confirmed these previous results as the IC_50_ was 74.13 ± 1.1 µg/ml which meant a potent effect compared with the IC_50_ of acarbose (the reference compound), which was 28.10 ± 0.7 µg/ml. The hydrolysis of lipids by pancreatic lipase into glycerol and fatty acids is well known. As a result, inhibiting this enzyme prevents lipids from being converted into absorbable compounds in the body. As a result, lipase inhibitory medicines or herbal items can be used to manage obesity [54]. *O. basilicum* essential oil anti-lipase activity was tested using orlistat as a reference control because of its similar mode of action. As stablished in other studies, *O. basilicum* essential oil significantly lowered both plasma triglycerides and cholesterol in acute hyperlipidemia induced by Triton WR-1339 in rats [28]. Irondi et al. examined the ethyl acetate leaf extract of *O. basilicum*. The extract exhibited excellent lipase inhibitory action, but it was lower compared to orlistat [56]. A similar finding was reported by Noor et al. who discovered that extracts of *O. basilicum* flowers and leaves possess significant lipase inhibitory action [54]. This recent study also showed an anti-lipase result in which the IC_50_ was 112.20 ± 0.7 µg/ml which can be described as a moderate effect compared with the IC_50_ of orlistat, which was 12.30 ± 0.8 µg/ml.

Antimicrobial resistance has recently been an issue in global healthcare systems, particularly in hospitals, and can impact anybody, anywhere, at any age. Many infectious disorders caused by fungus, viruses, parasites, and bacteria are at risk because of antimicrobial resistance [57, 58]. In fact, MRSA, *K. pneumoniae*, *E. coli*, *S. aureus*, *P. mirabilis*, and *P. aeruginosa* bacterial species can cause very harmful and lethal infectious diseases and have resistance to the most commonly used antibiotics currently [59, 60]. In addition, the most prevalent isolated yeast from bloodstream infections is *C. albicans*. This infection remains a serious problem in intensive care units all around the world despite impressive advancements in diagnostic and treatment techniques. This fungus species has resistance to the majority of currently utilized antifungal commercial drugs [61].

As a result, the healthcare system has recently turned to the use of phytomedicines to treat microbial diseases. The results of the antimicrobial test demonstrated that the essential oil of *O. basilicum* had a broad-spectrum antibacterial activity and suppressed the development of all tested microbial strains, in contrast to the antimicrobial medications ampicillin, ciprofloxacin, and fluconazole. The oil showed a range of minimum inhibitory concentrations (MICs) between 1 and 2.3 µg/mL for all tested bacterial strains. Intriguingly, the essential oil suppressed the development of the fungus strain *C. albicans* far more than the prospective antifungal medication fluconazole, with a MIC of 1.3 µg/ml. These results were consistent with Joshi (2014), who measured the minimal bactericidal concentration against different microbial strains and found that *O. basilicum* essential oil was active against gram-positive, gram-negative bacteria, and fungi [42]. The majority of essential oils tested for their antibacterial qualities had a more dramatic effect on gram-positive bacteria than other types of bacteria. The resistance of gram-negative bacteria to essential oil has been attributed to their hydrophilic outer membrane, which can prevent hydrophobic chemicals from penetrating the target cell membrane [62]. The presence of phenolic components in the essential oil may contribute to its antibacterial action by triggering intracellular ATP and potassium ion leakage, which results in cell death [63, 64].

Drug resistance against cancer is still a significant barrier in medical oncology. Resistance can develop clinically either before or after cancer treatment. Due to the fact that using natural plant products alone or in combination with chemotherapeutics may reduce resistance or limit the proliferation of cancer cells [65].

The anticancer activity of *O. basilicum* essential oil was tested against three kinds of cancer cells, namely HeLa, Hep3B, and MCF-7 cancer cells. Cervical cancer tissues were used to get HeLa cells, which are the most frequent kind of cancer in women [66]. This form of cancer can be caused by a variety of factors, including smoking, oral contraceptives, and HPV, which is a sexually transmitted infection [67]. Even though screening, diagnosis, and immunization programs have improved over the last few decades, this kind of cancer is still not under control. Breast cancer, one of the most common cancers in women, is the source of the MCF-7 cell line, which has a high death rate around the globe [68]. One of the most prominent risk factors for this form of cancer is estrogen [69]. Other risk factors, such as a history of the disease in one’s family, may also be significant. Cancer of the liver, or hepatocellular carcinoma, gave rise to the Hep3B cells. Hepatitis B virus (HBV) and hepatitis C virus (HCV) infections are the most common causes of hepatocellular cancer. Hepatomas develop as a result of the altered liver matrix caused by these diseases [70].

*O. basilicum* essential oil showed significant cytotoxic activity against liver cancer (Heb3B) (IC_50_ = 56.23 ± 1.32 µg/ml) and breast cancer (MCF7) (IC_50_ = 80.35 ± 1.17 µg/ml) cell lines when compared with doxorubicin (IC_50_ = 21.37 ± 0.62 and 15.02 ± 0.72 µg/ml respectivly). The obtained results were confirmed by many studies involving a variety of basil extracts and their anticancer effects on different cancer cell lines. Elansary and Mahmoud compared the anticancer activity of several compositions on multiple human cancer lines to see which combination had the highest effect. The *O. basilicum* species had the most potent anticancer effects among the investigated concentrations and varieties. All basil extracts promoted apoptosis and cell cycle progression, resulting in antiproliferation and cell death [71]. The presence of bioactive substances, such as rosmarinic, chicoric, and caftaric acids, was suggested to be associated with these properties [71, 72].

## Conclusion

For the first time, numerous biological investigations of the Palestinian *O. basilicum* seed essential oil were carried out in this study. The findings demonstrated that it has potent antioxidant, anti-α-glucosidase, and antimicrobial effects against gram-positive and gram-negative bacteria, as well as *C. albicans* fungus. In addition, it demonstrated possible cytotoxic activity against the cancer cell lines MCF-7 and Hep3B. These results showed that the essential oil of *O. basilicum* might be a significant source of natural antioxidants that are potentially effective in the detoxification mechanisms of living organisms, particularly against illnesses caused by oxidative stress. Additionally, it might be utilized as a complementary and alternative treatment for infectious disorders caused by pathogens. Consequently, this essential oil’s potential usage as a beneficial novel medicinal agent with functional properties for food, food supplements, and pharmaceutical items should be investigated.

## Data Availability

The datasets used and/or analysed during the current study are available from the corresponding author on reasonable request.
